# Transdermal bisoprolol for prevention of postoperative atrial fibrillation: A systematic review and meta‐analysis

**DOI:** 10.1002/joa3.13049

**Published:** 2024-05-02

**Authors:** Andrew G. Kim, Sandeep Banga, Qi Xuan Ang, Lalitsiri Atti, Harith Ghnaima, Saif AlAttal, Preeti Banga, Matthew D. Wilcox

**Affiliations:** ^1^ Internal Medicine Michigan State University East Lansing Michigan USA; ^2^ Cardiology, Michigan State University East Lansing Michigan USA; ^3^ Radiology University of Michigan Ann Arbor Michigan USA

## Abstract

**Background:**

The transdermal patch of bisoprolol available in Japan has been reported to demonstrate superior efficacy in preventing postoperative atrial fibrillation, possibly surpassing its oral counterpart. However, there has been no systematic review and meta‐analysis assessing the efficacy of transdermal bisoprolol.

**Methods:**

A comprehensive systematic literature search was conducted on PubMed, Embase, and Cochrane to identify all relevant studies assessing the efficacy of transdermal bisoprolol in preventing postoperative atrial fibrillation. The search covered studies from inception up to December 4, 2023. For data analysis, Review Manager (RevMan) 5.4 software was employed, using a random‐effects model to calculate risk ratios (RR) and 95% confidence intervals (CI).

**Results:**

Three studies, comprising a total of 551 patients (transdermal bisoprolol 228 and control 323), were included. There was a decreased risk of postoperative atrial fibrillation or atrial tachyarrhythmias in patients treated with transdermal bisoprolol (RR 0.43, 95% CI 0.27–0.67, *p* = .0002, *I* ^2^ = 0%).

**Conclusion:**

Transdermal administration of bisoprolol has consistently shown efficacy, and this pooled analysis supports its effectiveness. The heterogeneity of the included studies limits certain interpretations. Future randomized clinical trials may elucidate the superiority of transdermal administration over oral administration.

## Graphical abstract

Transdermal bisoprolol effectively reduces postoperative atrial fibrillation, potentially outperforming oral formulations.

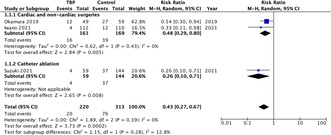

## 1 INTRODUCTION

Postoperative atrial fibrillation (POAF) is observed in 5%–60% of postsurgical patients, with a higher incidence following cardiac and valve surgeries.^1^ Although many cases spontaneously convert to normal sinus rhythm,^2^ POAF is linked to adverse outcomes, such as stroke and death.^3^

Beta‐blockers have been employed to prevent POAF in both cardiac^4^ and noncardiac surgeries.^5^ Bisoprolol, a selective beta‐1 receptor blocker without intrinsic sympathomimetic activity, not only controls heart rate but also exhibits antiarrhythmic effects in atrial fibrillation, potentially through the suppression of sympathetic activity.^6^ Recent advances in electrophysiology, such as cardioneuroablation, underscore the role of steady autonomic nervous system regulation in suppressing atrial fibrillation.^7^ In light of these findings, hypothesizing that maintaining a more stable serum beta‐blocker concentration would offer superior POAF prevention seems plausible.

Transdermal bisoprolol patch (TBP) (Bisono^®^ Tape; Toa Eiyo, Tokyo, Japan) offers advantages over oral bisoprolol fumarate (OBF) by ensuring a steady serum concentration. This could be beneficial in POAF prevention, considering that minimizing autonomic variability through autonomic denervation reduces atrial fibrillation recurrences.^7^ Hypothetically, TBP may outperform OBF in preventing POAF by potentially minimizing autonomic variability by maintaining steadier serum concentrations. Indeed, Okamura et al. (2019)^8^ demonstrated the superiority of the transdermal formula over the oral formula in preventing POAF. In this context, we conducted a comprehensive literature review on the efficacy of TBP.

## 2 METHODS

### 2.1 Search strategy

An extensive search of the literature was conducted using the databases PubMed, Embase, and Cochrane, gathering all studies available from their start dates up to December 4, 2023. The search terms used included “bisoprolol,” “transdermal,” and “atrial fibrillation,” in various word forms, applied across all texts.

### 2.2 Study selection

**FIGURE 1:**
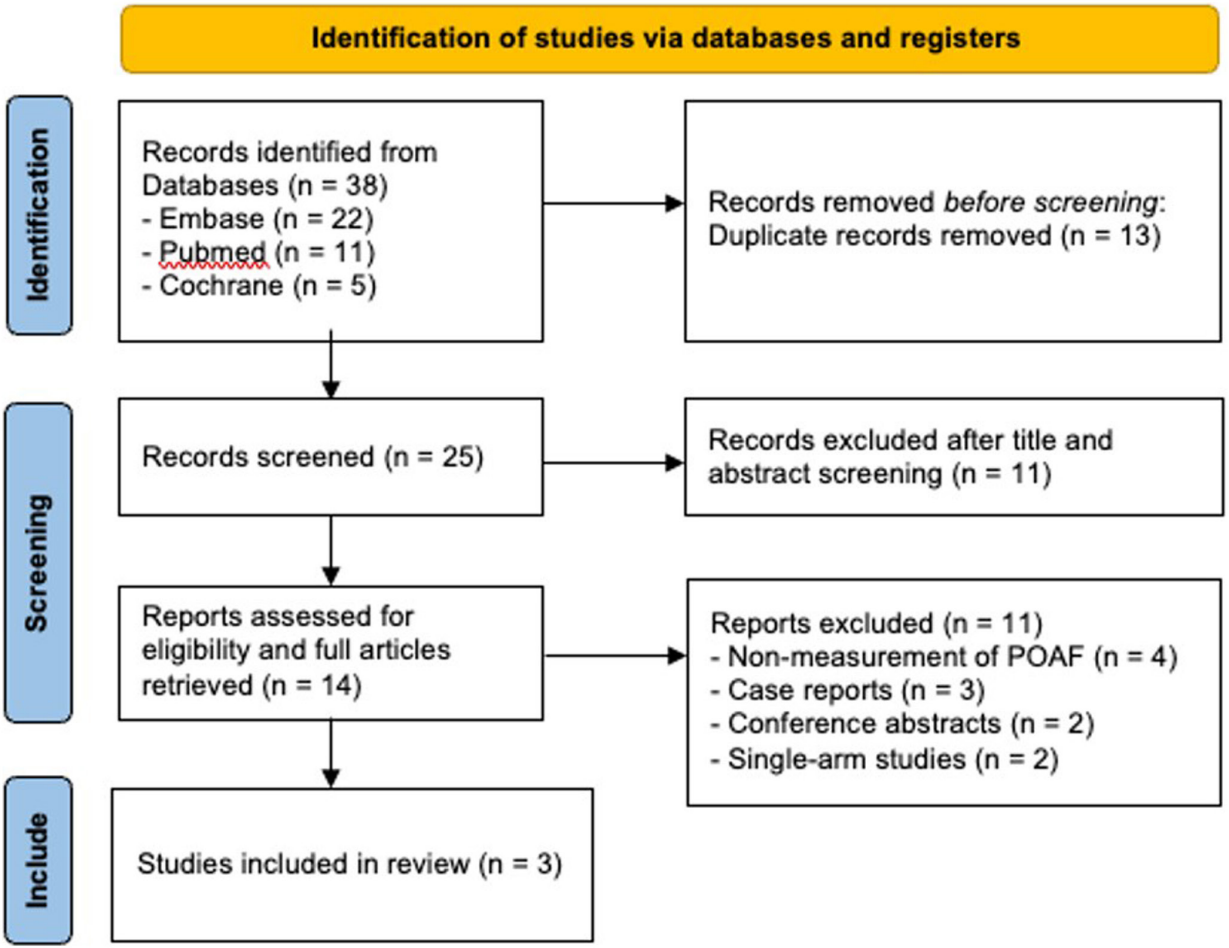
Preferred reporting items for systematic reviews and meta‐analysis (PRISMA) flowchart.

Two reviewers (AGK and SB) independently conducted a thorough evaluation of the study articles. We included all studies that evaluated the efficacy of TBP in preventing POAF with a comparison group. Case reports or abstracts with insufficient data were excluded, along with single‐arm studies that only evaluated the efficacy of TBP. Five studies^8^, ^9^, ^10^, ^11^, ^12^ from Japan evaluating the effect of TBP in POAF were retrieved. Three studies^8^, ^9^, ^10^ were included in the review. Two studies^11^, ^12^ only included patients who received TBP, thereby being removed from the comparative analysis. The meta‐analysis was performed following the Preferred Reporting Items for Systematic Reviews and Meta‐Analysis (PRISMA) guidelines (Figure 1).

### 2.3 Statistical analysis

The pooled analysis was conducted using Review Manager (RevMan) 5.4 software, applying a random‐effects model for the calculation of risk ratios (RRs) and 95% confidence intervals (CIs). Statistical significance was determined by an overall *p*‐value below .05. The *I* ^2^ test was used to evaluate heterogeneity.

## 3 RESULTS

### 3.1 Study characteristics

The analysis comprised one multicenter randomized controlled study^9^ and two single‐center retrospective studies,^8^, ^10^ with a total of 551 patients—228 in the TBP arm and 323 in the control arm. Heterogeneity existed among the three studies in terms of the type of surgery, control group, and outcome measures. Detailed characteristics of the included studies and patients are listed in Tables 1, 2.

**TABLE 1:** Characteristics of the included studies.

Author	Okamura et al. (2019)^8^	Iwano et al. (2021)^9^	Suzuki et al. (2021)^10^
Country	Tokyo, Japan	Okayama, Japan	Akashi, Japan
Study design	Single‐center retrospective study	Multicenter randomized controlled study	Single‐center retrospective study
Study period	April 2016 to February 2018	November 2014 to February 2019	January 2018 to June 2019
Sample size, N	Total 108; TBP 49 and control (OBF) 59	Total 240; TBP 120 and control 120 at randomization (intention‐to‐treat analysis) Total 222; TBP 112 and control 110	Total 203; TBP 59 and control 144
Population	Patients who underwent cardiac and/or thoracic aortic surgery	Patients aged ≥60 years, with hypertension and a Revised Cardiac Risk Index (RCRI) score ≥2, scheduled for noncardiac surgery, and not receiving beta‐blockers. Patients with chronic AF were excluded.	Patients with paroxysmal atrial fibrillation who underwent their first ablation and had a left ventricular ejection fraction of ≥35%
Intervention	TBP 4 mg (equivalent to OBF 2.5 mg) or lower daily from POD 1	TBP 4 mg daily or lower from 7 days before surgery until POD 7	TBP 4 mg or dose adjusted
Comparison	No TBP; OBF 2.5 mg or lower daily from POD 1	No TBP; no beta‐blockade was given to the comparison group	No TBP; oral antiarrhythmics were substituted, including OBF (32.6%), carvedilol (15.3%), atenolol (1.4%), verapamil (1.4%), bepridil (2.8%), flecainide (11.1%), and amiodarone (0.7%)
Outcome	Postoperative AF	Postoperative AF	Postprocedural early recurrences of AF or AT
Definition of outcome	New onset of postoperative AF lasting >30 seconds recorded using a telemetry system throughout hospitalization	Onset of AF that occurred within 30 days after surgery	Any episode of AF or AT lasting ≥30 seconds and occurring within 90 days of AF ablation
Methods of AF detection	Telemetry system throughout hospitalization with average postoperative 23 ± 14 days	ECG monitoring for several days after surgery. After the ECG monitoring was discontinued, arrhythmia was detected on the basis of the patient's symptoms and vital signs	ECG monitor, 12‐lead ECG, Holter ECG, or CIED
Adjusted confounders	– Age (*p* = .76), gender (*p* = .44), BMI (*p* = .28), NYHA class 3 or 4 (*p* = .61), DM (*p* = .25), dyslipidemia (*p* = .81), IHD (*p* = 1.00), stroke (*p* = .54), COPD (*p* = .59), CKD (*p* = .75), hemodialysis (*p* = 1.00), LVEF (*p* = .87), LA diameter (*p* = .27), and BNP (*p* = .68) – Preoperative medication: ACEI (*p* = 1.00), ARB (*p* = .39), CCB (*p* = .85), statin (*p* = .38), beta‐blocker (*p* = .16), and aldosterone blocker (*p* = 0.69) – Type of surgery: isolated valve surgery (*p* = 0.84), isolated CABG (*p* = 1.00), valve surgery + CABG (*p* = 1.00), isolated aortic surgery (*p* = .85), aortic surgery + valve surgery (*p* = .45), others (*p* = .63), cardiopulmonary bypass time (*p* = .19), and aortic cross‐clamp time (*p* = .70)	Toda et al. (2020) (MAMACARI trial)^13^ – Age, gender, BMI, RCRI score, high‐risk procedure, history of IHD, history of heart failure, history of cerebrovascular disease, renal failure, preoperative insulin use, ASA class 2, ASA class 3, hypertension, DM, dyslipidemia, smoker, paroxysmal AF, PAD, obstructive lung disease, and asthma – Previous treatment: PCI, CABG, and pacemaker – Systolic blood pressure, diastolic blood pressure, pulse rate, LVDF, LA diameter, E/e’, and eGFR – Medication: CCB, ACEI, ARB, diuretics, antiplatelet therapy, oral anticoagulants, and statin – Vital capacity, FEV1 – Surgical specialty: general, thoracic, vascular, neurosurgery, orthopedic, otolaryngology, urology, and others – Anesthesia: inhalation anesthesia and duration of surgery	– Age (*p* = .91), gender (*p* = .13), BMI (*p* = .45), smoking (*p* = .76), duration of AF (0.41), hypertension (*p* = .84), DM (*p* = .99), hyperlipidemia (*p* = .16), stroke (*p* = .55), HF (*p* = .56), CAD (*p* = .19), valvular heart disease (*p* = .52), DCM (*p* = .71), HCM (*p* = .50), pacemaker (*p* = .51), ICD/CRT (*p* = .71), ILR (*p* = .12), LA diameter (*p* = .31), LVEF (*p* = .90), LCPV (p = .55), BUN (*p* = .08), creatinine (*p* = .96), eGFR (*p* = .59), hemoglobin (*p* = .20), and BNP (*p* = .11) – Medications on discharge: verapamil (*p* = .50), bepridil (*p* = .25), amiodarone (*p* = .50), and ACEI/ARB (*p* = .94) – Ablation procedure: CTI ablation (*p* = .92), additional procedure (*p* = .83), posterior box (*p* = .74), roof line (*p* = .42), bottom line (*p* = .20), mitral isthmus line (*p* = .50), SVC isolation (*p* = .50), non‐PV trigger ablation (*p* = .67), and total duration of procedure (*p* = .17)
Covariates in model	Hypertension (TBP 76%, control 56%, *p* = .04)		– Medications on discharge: beta‐blocker (TBP 100%, control 49.3%, *p* < .0001), OBF (TBP 0%, control 32.6%, *p* < .0001), carvedilol (TBP 0%, control 15.3%, *p* = .001), flecainide (TBP 0%, control 11.1%, *p* = .003), and statin (TBP 35.6%, control 21.5%, *p* = .037) – Ablation procedure: cryoballoon (TBP 50.8%, control 66%, *p* = .044)

Abbreviations: ACEI, angiotensin‐converting enzyme inhibitor; AF, atrial fibrillation; ARB, angiotensin II receptor blocker; ASA, American Society of Anesthesiologists; AT, atrial tachycardia; BNP, B‐type natriuretic peptide; BMI, body mass index; BUN, blood urea nitrogen; CABG, coronary artery bypass grafting; CAD, coronary artery disease; CCB, calcium channel blocker; CIED, cardiac implantable electronic device; CKD, chronic kidney disease; COPD, chronic obstructive pulmonary disease; CTI, cavotricuspid isthmus; DCM, dilated cardiomyopathy; DM, diabetes mellitus; eGFR, estimated glomerular filtration rate; E/e’, ratio of early transmitral flow velocity to early diastolic mitral annular velocity; FEV1, forced expiratory volume in 1 s; HCM, hypertrophic cardiomyopathy; HF, heart failure; ICD/CRT, implantable cardioverter‐defibrillator/cardiac resynchronization therapy; IHD, ischemic heart disease; ILR, implantable loop recorder; LA, left atrium; LCPV, left common pulmonary vein; LVEF, left ventricular ejection fraction; LVDF, left ventricular diastolic function; NYHA, New York Heart Association; OBF, oral bisoprolol fumarate; PAD, peripheral arterial disease; PCI, percutaneous coronary intervention; POD, postoperative day; RCRI, revised cardiac risk index; SVC, superior vena cava; TBP, transdermal bisoprolol patch.

**TABLE 2:** Baseline characteristics of study patients.

	Okamura et al. (2019)^8^		Iwano et al. (2021),^9^ Toda et al. (2020)^13^		Suzuki et al. (2021)^10^	
	TBP (*n* = 49)	Control (*n* = 59)	TBP (*n* = 120)	Control (*n* = 120)	TBP (*n* = 59)	Control (*n* = 144)
Age (years)	69.2 ± 14.5	68.4 ± 14.0	76 (67–85)	76 (66–86)	66.9 ± 12.7	66.7 ± 10.0
Male gender	25 (51%)	25 (42%)	92 (77%)	92 (77%)	38 (64.4%)	108 (75%)
BMI (kg/m^2^)	22.3 ± 3.7	23.2 ± 4.7	22.8 (20.6–24.7)	22.8 (21.2–25.3)	23.5 ± 3.6	23.9 ± 3.6
Hypertension	37 (76%)	33 (56%)	120 (100%)	120 (100%)	30 (50.8%)	71 (49.3%)
DM	4 (8%)	10 (17%)	53 (44%)	62 (52%)	9 (15.3%)	22 (15.3%)
Heart failure	17 (35%)	23 (39%)	34 (28%)	29 (24%)	2 (3.4%)	4 (2.8%)
Dyslipidemia	9 (18%)	13 (22%)	62 (52%)	62 (52%)	20 (33.9%)	35 (24.3%)
IHD	9 (18%)	10 (17%)	73 (61%)	65 (54%)	6 (10.2%)	8 (5.6%)
Stroke	6 (12%)	5 (8%)	37 (31%)	36 (30%)	4 (6.8%)	9 (6.3%)
LVEF (%)	61.4 ± 12.3	62.0 ± 13.9	66 (61–69)	66 (62–69)	66.0 ± 6.5	65.9 ± 6.9
LA diameter (mm)	37 ± 9	40 ± 7	37 (34–40)	36 (32–40)	36.4 ± 4.6	35.6 ± 5.3
BNP (pg/mL)	330 ± 607	282 ± 491	–	–	99.2 ± 19.7	64.9 ± 8.1
Beta‐blocker	13 (27%)^a^	9 (15%)^a^	0 (0%)^a^	0 (0%)^a^	59 (100%)	71 (49.3%)
ACEI or ARB	18 (36%)	26 (45%)	71 (67%)	82 (68%)	20 (33.9%)	48 (33.3%)
CCB	21 (43%)	24 (41%)	71 (59%)	70 (58%)	–	–
Statin	10 (20%)	17 (29%)	44 (37%)	49 (41%)	21 (35.6%)	31 (21.5%)

Abbreviations: ACEI, angiotensin converting enzyme inhibitor; ARB, angiotensin II receptor blocker; BMI, body mass index; BNP, B‐type natriuretic peptide; CCB, calcium channel blocker; DM, diabetes mellitus; IHD, ischemic heart disease; LA, left atrium; LVEF, left ventricular ejection fraction; POD, postoperative day; TBP, transdermal bisoprolol patch.

^a^ Preoperative medication prior to admission.

#### 3.1.1 Cardiac and noncardiac surgeries

Okamura et al. (2019)^8^ conducted a single‐center retrospective study in Tokyo, Japan, from April 2016 to February 2018, involving 108 participants undergoing cardiac/thoracic aortic surgery. The study compared TBP 4 mg or lower from POD 1 to discharge to no TBP (OBF 2.5 mg or lower), with 49 participants in the TBP arm and 59 in the control (OBF) arm. The outcome measured was the new onset of AF (>30s).

Iwano et al. (2021),^9^ a subanalysis of the MAMACARI trial with baseline characteristics stated in Toda et al. (2020),^13^ is a multicenter randomized controlled study conducted in Okayama, Japan, from November 2014 to February 2019. It included 240 participants aged ≥60 with hypertension and a revised cardiac risk index score ≥2 scheduled for noncardiac surgery. Patients receiving beta‐blockers and patients with chronic AF were excluded. The study compared TBP 4 mg or lower from 7 days pre‐surgery to POD 7 to no TBP, with 120 participants in each arm. The measured outcome was AF onset within 30 days postsurgery.

#### 3.1.2 Catheter ablation

Suzuki et al. (2021)^10^ is a single‐center retrospective study in Akashi, Japan, from January 2018 to June 2019, involving 203 participants with paroxysmal AF undergoing their first ablation, with LVEF ≥35%. The study compared TBP 4 mg or dose adjusted to no TBP immediately after AF ablation for 3 months, with 59 participants in the TBP arm and 144 in the control arm. The control arm could have received either OBF or other antiarrhythmics instead of TBP, including OBF (32.6%), carvedilol (15.3%), atenolol (1.4%), verapamil (1.4%), bepridil (2.8%), flecainide (11.1%), and amiodarone (0.7%). The measured outcome was early recurrences of atrial arrhythmias within 90 days post‐AF ablation (blanking period).

### 3.2 Outcomes

#### 3.2.1 Cardiac and noncardiac surgeries

The combined analysis by Okamura et al. (2019),^8^ which evaluated postoperative patients who underwent cardiac or thoracic aortic surgery, and Iwano et al. (2021),^9^ which assessed those undergoing noncardiac surgeries, showed a decreased risk of POAF in the TBP group; risk ratio 0.48, 95% CI 0.29–0.80, *p* = .005, *I* ^2^ = 0%.

#### 3.2.2 Catheter ablation

Suzuki et al. (2021),^10^ which evaluated patients who underwent catheter‐guided AF ablation procedures, revealed a decreased risk of atrial arrhythmias in the TBP group; risk ratio 0.26, 95% CI 0.10–0.71, *p* = .008.

#### 3.2.3 Overall

**FIGURE 2:**
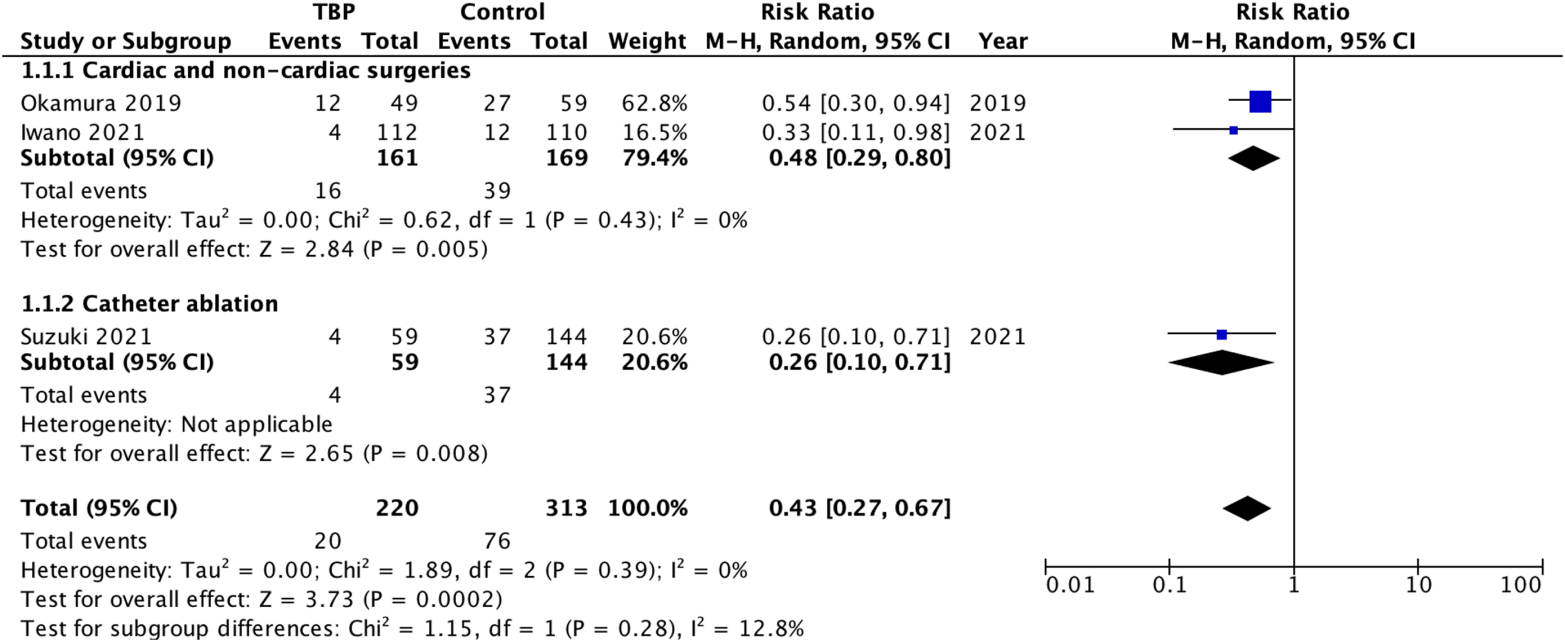
Forest plot of outcomes. CI, confidence interval; M‐H, Mantel‐Haenszel; TBP, transdermal bisoprolol patch.

The pooled analysis of both subgroups revealed a decreased risk of either postoperative atrial fibrillation or atrial tachyarrhythmias in patients treated with transdermal bisoprolol, risk ratio 0.43, 95% CI 0.27–0.67, *p* = .0002, *I* ^2^ = 0%. Despite heterogeneity of the three studies in terms of type of surgery/procedure and comparison group, statistical heterogeneity of the outcome between the TBP arm and the control arms was 0%. In the test for subgroup differences between cardiac and noncardiac surgeries versus catheter ablation, the heterogeneity (*I* ^2^) was 12.8%, indicating low variability among the subgroups. Each study yielded statistically and clinically significant results, as illustrated in Figure 2.

## 4 DISCUSSION

Postoperative atrial fibrillation (POAF) poses a clinical challenge, affecting a notable percentage of postsurgical patients, especially after cardiac and valve surgeries.^1^ While many cases spontaneously revert to normal sinus rhythm, the association between POAF and adverse outcomes, including stroke and death, underscores the need for effective prevention strategies.^2^, ^3^

Bisoprolol, a beta‐1 receptor blocker without intrinsic sympathomimetic activity, is commonly used for ventricular rate control in atrial fibrillation. It also demonstrates an antiarrhythmic effect, likely achieved by suppressing sympathetic activity, particularly in maintaining sinus rhythm for patients with a history of atrial fibrillation mediated by sympathetic tone.^6^

Evidence suggests that minimizing autonomic variability through vagal and sympathetic denervation is associated with a reduction in atrial fibrillation recurrences.^7^ This insight guides our exploration of the potential benefits of maintaining a more stable blood bisoprolol concentration through transdermal administration. Our study supports the advantages of transdermal bisoprolol patch (TBP) in preventing POAF.

### 4.1 Study limitations

Our study's interpretability is hindered by the variability in surgical procedures, control groups, and outcome measures across the included studies. This variability affects the consistency of the findings and their applicability to different surgical contexts.

The study included both cardiac and noncardiac surgeries, as reported in studies by Okamura et al. (2019)^8^ and Iwano et al. (2021),^9^ as well as AF ablation procedures, according to Suzuki et al. (2021).^10^ This mix of different interventions may influence the generalizability of the results, despite the consistency observed.

Okamura et al. (2019)^8^ conducted a direct comparison between the TBP group and the OBF group. In contrast, Suzuki et al. (2021)^10^ compared the TBP group with a control group, which included a significant portion of patients receiving alternative oral antiarrhythmics. These included OBF (32.6%), carvedilol (15.3%), atenolol (1.4%), verapamil (1.4%), bepridil (2.8%), flecainide (11.1%), and amiodarone (0.7%).

Iwano et al. (2021),^9^ however, did not compare TBP with OBF but instead evaluated TBP against no beta‐blocker treatments. Despite these discrepancies, all included studies consistently demonstrated the efficacy of TBP in preventing POAF. Both Okamura et al. (2019)^8^ and Suzuki et al. (2021)^10^ suggested TBP's superiority over OBF in this context.

## 5 CONCLUSION

In conclusion, our study supports the potential of transdermal bisoprolol as a superior preventive measure against postoperative atrial fibrillation (POAF). The sustained suppression of sympathetic tone through the transdermal patch may introduce a clinically relevant avenue for exploration. Future randomized clinical trials may elucidate the nuanced efficacy of transdermal bisoprolol over oral bisoprolol in diverse surgical and procedural contexts.

## CONFLICT OF INTEREST STATEMENT

The authors have no competing interests to declare.
